# QSAR-driven rational design of novel DNA methyltransferase 1 inhibitors 

**DOI:** 10.17179/excli2020-1096

**Published:** 2020-04-02

**Authors:** Chuleeporn Phanus-umporn, Veda Prachayasittikul, Chanin Nantasenamat, Supaluk Prachayasittikul, Virapong Prachayasittikul

**Affiliations:** 1 Center of Data Mining and Biomedical Informatics, Faculty of Medical Technology, Mahidol University, Bangkok 10700, Thailand; 2 Department of Clinical Microbiology and Applied Technology, Faculty of Medical Technology, Mahidol University, Bangkok 10700, Thailand

**Keywords:** DNA methyltransferase 1, QSAR, computer-aided drug design, rational design, structural modification, epigenetic modulators

## Abstract

DNA methylation, an epigenetic modification, is mediated by DNA methyltransferases (DNMTs), a family of enzymes. Inhibitions of these enzymes are considered a promising strategy for the treatment of several diseases. In this study, a quantitative structure-activity relationship (QSAR) modeling was employed to understand the structure-activity relationship (SAR) of currently available non-nucleoside DNMT1 inhibitors (i.e., indole and oxazoline/1,2-oxazole scaffolds). Two QSAR models were successfully constructed using multiple linear regression (MLR) and provided good predictive performance (R^2^_Tr _= 0.850-0.988 and R^2^_CV _= 0.672-0.869). Bond information content index (BIC1) and electronegativity (R6e+) are the most influential descriptors governing the activity of compounds. The constructed QSAR models were further applied for guiding a rational design of novel inhibitors. A novel set of 153 structurally modified compounds were designed *in silico *according to the important descriptors deduced from the QSAR finding, and their DNMT1 inhibitory activities were predicted. This result demonstrated that 86 newly designed inhibitors were predicted to elicit enhanced DNMT1 inhibitory activity when compared to their parent compounds. Finally, a set of promising compounds as potent DNMT1 inhibitors were highlighted to be further developed. The key SAR findings may also be beneficial for structural optimization to improve properties of the known inhibitors.

## Introduction

Epigenetics is an alteration of gene expression without changing the genomic structure. Epigenetics machinery includes DNA methylation, histone modification (e.g., acetylation and methylation) and non-coding RNAs (Handy et al., 2011[22]). Epigenetic regulation can be altered by exogenous factors such as diet and exposing environment (Kanherkar et al., 2014[28]). Thus, epigenetic alteration serves as dynamic flexible responses which can be reversibly modified throughout the lifetime (Schuebel et al., 2016[51]). Epigenetic regulation has been recognized to play an important role in driving cells toward normal cellular phenotypes and functions. An alteration of epigenetic regulation also has been noted in pathogenesis of many diseases including cardiovascular diseases (Abi Khalil, 2014[2]), neurological diseases (Landgrave-Gómez et al., 2015[31]), metabolic disorders (Kuneš et al., 2015[30]) and cancers (Verma, 2013[55]). Among all, DNA methylation is considered to be one of the most common modifications found in many diseases (Jin and Liu, 2018[27]). 

DNA methylation is a process by which a methyl group (-CH_3_) is transferred from S-adenosyl-L-methionine (SAM) to the C-5 position of cytosine residue of CpG islands, which are regions of large repetitive CpG dinucleotides (Wang and Leung, 2004[56]). This reaction requires a key catalytic enzyme namely DNA methyltransferases (DNMTs). DNMTs are categorized into 5 types i.e., DNMT1, DNMT2, DNMT3A, DNMT3B and DNMT3L (Zhang and Xu, 2017[62]). However, DNMT1 is considered to be the most stable epigenetic mark and is abundantly found in human cells (Hermann et al., 2004[24]). DNMT1 acts as a maintenance methyltransferase which functions to control level of gene expressions by inhibiting gene transcription leading to gene silencing (Heerboth et al., 2014[23]). An aberration of DNMT1 function (either hyper- or hypo-methylation) has been observed for many diseases. For example, an alteration of DNMT1 leads to an inactivation of several key genes in cancer cells (Esteller, 2008[15]; Lee et al., 2010[32]; Xu et al., 2011[59]). Excessive DNMT1 activity and hypermethylation are also found in many neurological disorders (Wüllner et al., 2016[58]; Yokoyama et al., 2017[60]). Notably, these diseases are multifactorial disorders in which exogenous factors play crucial roles. Along with its reversible nature, a modification of DNMT1 activity serves as an attractive treatment strategy toward these diseases.

Recently, several DNMT inhibitors (DNMTi) have been reported to successfully treat many diseases (e.g., breast cancer (Gupta et al., 2019[20]; Luo et al., 2018[35]), pancreatic carcinoma (Li et al., 2010[34]), Huntington's disease (Pan et al., 2016[40]), acute myeloid leukemia (Benetatos and Vartholomatos, 2016[5]), myelodysplastic syndrome (Stresemann et al., 2008[53]), sickle cell anemia (Fathallah and Atweh, 2006[17]; Saunthararajah et al., 2003[50]) and β-thalassemia (Ley et al., 1982[33])). In addition, a demethylation by DNMTi displayed preferable clinical outcome against chemoresistance cancer cells which are not responsive to standard chemotherapy (Clozel et al., 2013[11]). To date, two inhibitors (e.g., 5-azacitidine and decitabine) have been approved by the U.S. Food and Drug Administration (FDA) and European Medicines Agency for treatment of acute myeloid leukemia (AML) and myelodysplastic syndrome (MDS) (European Medicines Agency, 2009[16]; Nieto et al., 2016[39]; Saba, 2007[49]). Despite their high efficiency, these nucleoside inhibitors are unstable compounds with poor bioavailability and cytotoxicity (Erdmann et al., 2015[14]). The cytotoxicity has been noted to be derived from their mechanisms of action which incorporates into DNA and RNA of the cells. Therefore, there is a growing interest of developing alternative non-nucleoside inhibitors to avoid these limitations. Some non-nucleoside inhibitors are along the way of development, however, their potencies are still lower than those of nucleoside inhibitors and none of them have been approved for clinical uses (Chuang et al., 2005[10]; Valente et al., 2014[54]; Zhong et al., 2016[63]). Recently, quinoline scaffold has been reported to exhibit DNMT1 inhibitory activity (Zwergel et al., 2020[64]).

Indole and oxazoline are attractive scaffolds for drug discovery. Indole analogs have been reported to exhibit anticancer (Kumar et al., 2010[29]; Prakash et al., 2018[44]), antimicrobial (Hong et al., 2017[25]), antioxidant (Demurtas et al., 2019[12]) and anti-inflammatory (Abdellatif et al., 2016[1]; Rani et al., 2004[46]) activities. Oxazoline derivatives have been documented for their anticancer (Kumar et al., 201[29]0), antimicrobial (Zhang et al., 2011[61]), antioxidant (Parveen et al., 2013[41]) and antidiabetic (Ashton et al., 2005[4]) activities. Additionally, both of these derivatives have been reported to inhibit DNMT1 activity by reducing the affinity of the enzyme toward SAM competition (Asgatay et al., 2014[3]; Castellano et al., 2008[6], 2011[7]; Castillo-Aguilera et al., 2017[8]; Siedlecki et al., 2006[52]). 

Computational approaches have been recognized for their facilitating roles in drug development process (Prachayasittikul et al., 2015[43]). Quantitative structure-activity relationship (QSAR) is a method to find a relationship between chemical structures of compounds and their biological activities and is one of the most commonly used approaches to increase success rate and reduce time of drug development (Nantasenamat et al., 2009[37]). QSAR modeling reveals a set of key chemical features and physicochemical properties that are essential for potent activity which would be beneficial for guiding the structural design and optimization to obtain potential compounds with preferable properties (Prachayasittikul et al., 2015[43], 2017[42]; Pratiwi et al., 2019[45]; Worachartcheewan et al., 2020[57]).

**Figure 1:**
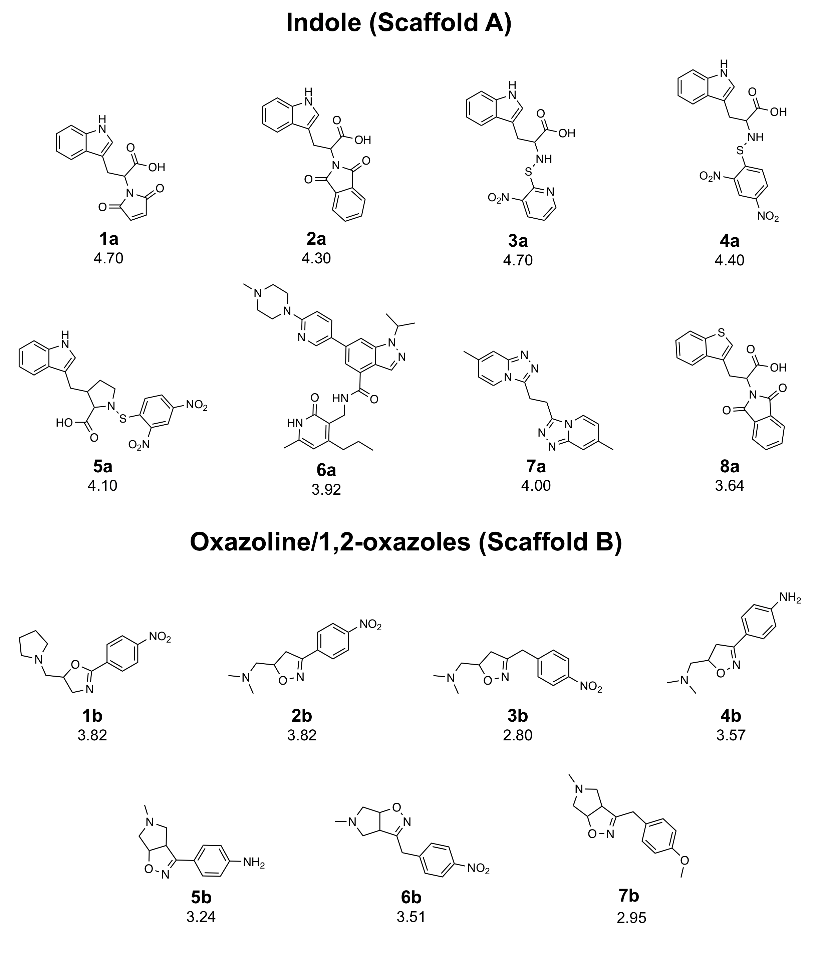
Chemical structures of DNMT1 inhibitors and their pIC_50_ values; scaffold A (indoles) and scaffold B (oxazoline and 1,2-oxazoles)

In this study, QSAR modeling was performed to reveal structure-activity relationship (SAR) of indole-based (scaffold A) and oxazoline/1,2-oxazole-based (scaffold B) DNMT1 inhibitors (Figure 1(Fig. 1)). Multiple linear regression (MLR) algorithm was used for model construction to allow effective SAR analysis. To expand structural diversity, an additional set of 153 structurally modified compounds were rationally designed according to key descriptors obtained from QSAR findings and their activities were predicted. SAR analysis was performed to gain insights toward essential key features required for potent activity. Additionally, chemical space plots were generated to illustrate drug-likeness of the studied compounds. Finally, a set of promising novel DNMT1 inhibitors were highlighted to be further developed. SAR findings also would be useful for screening, guiding design and structural optimization of the related compounds for DNMT1 inhibition.

## Materials and Methods

**Figure 2:**
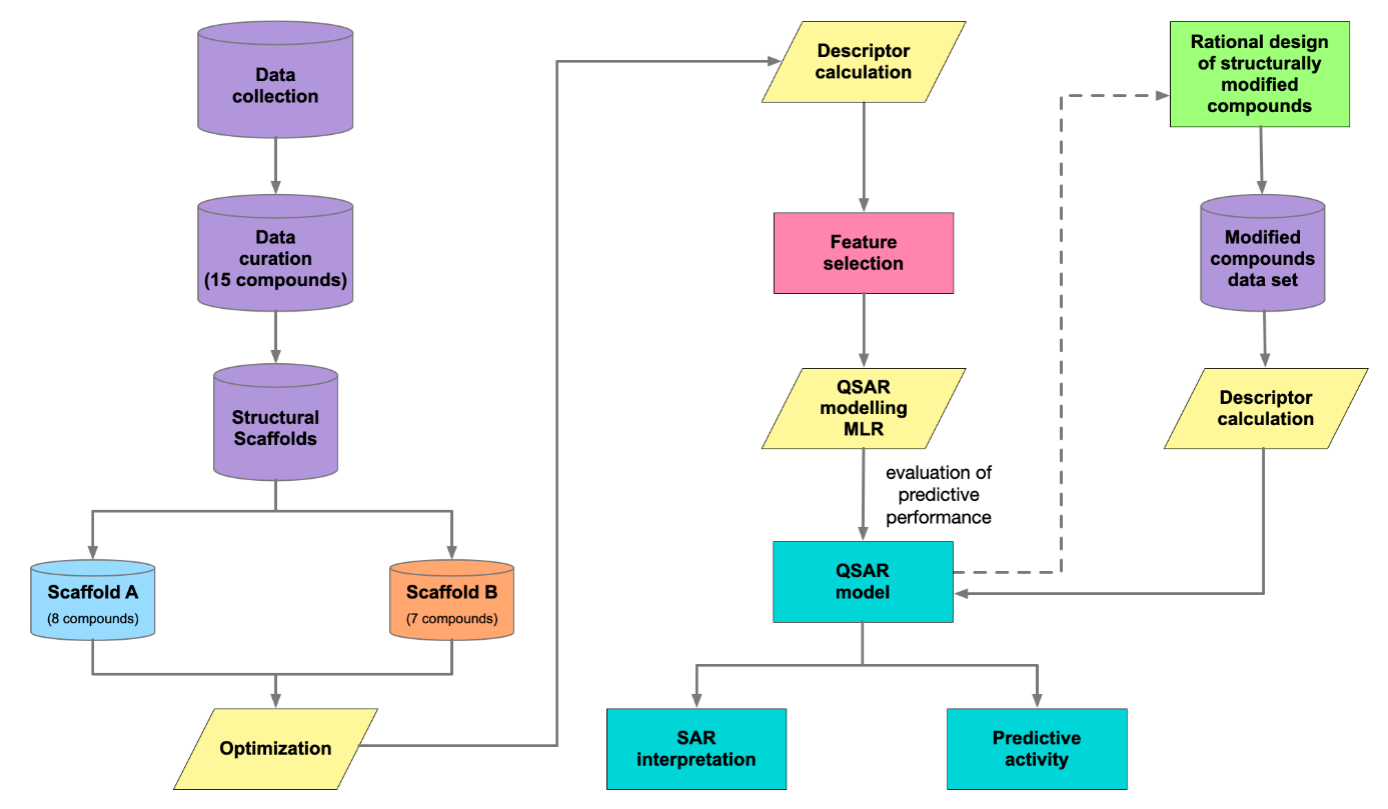
Workflow of QSAR modeling for investigating DNMT1 inhibitor

A schematic summary of QSAR modeling process is presented in Figure 2(Fig. 2).

### Data collection

A set of bioactive compounds with DNMT1 inhibitory activity were collected from ChEMBL25 database (EMBL-EBI, 2019[13]). The datasets have been thoroughly curated according to the established protocol (Fourches et al., 2010[18]). The main steps of data curation are as followed (i) removal of inorganics, salts and mixtures, (ii) structural validation and cleaning, (iii) normalization of specific chemotypes, (iv) deletion of duplicates and (v) final checking. As a result, a final data set of DNMT1 inhibitors, comprising chemical structures of 15 inhibitors (in SMILES format) and their bioactivity (IC_50_ values), was primarily compiled from 5 original articles (Asgatay et al., 2014[3]; Castellano et al., 2011[7]; Erdmann et al., 2015[14]; Siedlecki et al., 2006[52]; Valente et al., 2014[54]). Afterwards, these compounds were manually grouped according to their core structures into 2 groups i.e., scaffold A (indole derivatives) and scaffold B (oxazoline/1,2-oxazole derivatives), to obtain data sets consisted of 8 and 7 compounds belonging to scaffolds A (**1a**-**8a**) and B (**1b**-**7b**), respectively (Figure 1(Fig. 1)). Bioactivities of DNMT1 inhibitors (IC_50 _values) were converted to pIC_50_ values by taking the negative logarithm based 10.

### Geometry optimization

Chemical structures in SMILES format were converted into MOL format using molconvert (ChemAxon, 2018[9]). All compounds were geometrically optimized using Gaussian 09 software (Frisch et al., 2009[19]) to obtain low energy conformation by density functional theory (DFT) computation using Becke's three-parameter Lee-Yang-Parr hybrid functional (B3LYP) in concomitant with the LanL2DZ basis set.

### Molecular descriptors

Molecular descriptors are a set of numerical values representing the molecules in terms of their connectivity, constitution and physicochemical properties (Nantasenamat et al., 2010[38]). Two types of descriptors (i.e., quantum chemical descriptors and dragon descriptors) were used for QSAR modeling due to their interpretable nature. 

Optimized structures were extracted to obtain a set of 13 quantum chemical descriptors using an in-house developed script. A set of quantum chemical descriptors includes the total energy of the molecule, highest occupied molecular orbital (HOMO) energy, lowest unoccupied molecular orbital (LUMO) energy, dipole moment (μ), electron affinity (EA), ionization potential (IP), energy difference of HOMO and LUMO states (HOMO-LUMO), Mulliken electronegativity (χ), hardness (η), softness (S), electrophilicity (ω), electrophilic index (ωi), most negative atom in the molecule (Qneg), most positive atom in the molecule (Qpos) and the mean absolute atomic charge (Qm). Furthermore, optimized structured were used as input files for calculation of molecular descriptors using Dragon 5.5 software (Mauri et al., 2006[36]) to obtain a set of 3,224 molecular descriptors, comprising 22 categories: 48 constitutional descriptors, 119 topological descriptors, 47 walk and path counts, 33 connectivity indices, 47 information indices, 96 2D autocorrelation, 107 edge adjacency indices, 64 Burden eigenvalues, 21 topological charge indices, 44 eigenvalue-based indices, 41 randic molecular profiles, 74 geometrical descriptors, 150 RDF descriptors, 160 3D-MoRSE descriptors, 99 WHIM descriptors, 197 GETAWAY descriptors, 154 functional group counts, 120 atom-centred fragments, 14 charge descriptors, 29 molecular properties, 780 2D binary fingerprints and 780 2D frequency fingerprints.

### Feature selection

To reduce overfitting and improve accuracy of prediction, correlation-based feature selection was employed for selecting a set of informative descriptors. Initially, Pearson's correlation coefficient (r) values were calculated for each pair of descriptor and bioactivity (pIC_50_). A cutoff value of 0.8 was used to select an initial set of correlated descriptors (with |r| ≥ 0.8) for further selection with multiple linear regression (MLR) method using SPSS (IBM Corp., 2011[26]). 

As a result, final sets of informative descriptors were obtained for further QSAR modeling.

### QSAR model construction

MLR is one of the commonly used machine learning algorithms to reveal a linear relationship between a set of independent variables (i.e., molecular descriptors; *X_n_*) and the dependent variable of interest (i.e., DNMT1 inhibitory activity; *Y*). In this study, two QSAR models were separately constructed according to their distinct scaffolds (i.e., scaffolds A and B). For each input data set, a set of selected descriptor values of the compounds along with their bioactivities (pIC_50_ value) were provided to train the machine. MLR models were constructed using Weka software (Hall et al., 2008[21]) as shown in Equation 1:


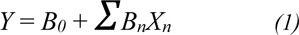


where *Y *is the pIC_50_ values of compounds, *B_0_* is the intercept and *B_n_* are the regression coefficient of descriptors *X_n_*.

### Model validation

The data set was divided into training set and testing set by leave-one-out cross validation (LOO-CV) (Roy et al., 2015[48]). N is the number of samples in the data set. One sample was removed from the whole data set to be predicted, whereas the remaining samples (N-1) were used as the training set. The same sampling process was continued until every sample was leaved out to be predicted as Y variable (activity).

### Evaluation of the predictive performance of QSAR model

Two statistical parameters such as correlation coefficient (R) and root mean square error (RMSE) values were calculated to assess the predictive performance of the constructed QSAR models (Prachayasittikul et al., 2017[42]; Pratiwi et al., 2019[45]).

### Prediction of modified compounds

A set of 153 structurally modified compounds were rationally designed according to key descriptors of the constructed QSAR models. All newly designed compounds were drawn, geometrically optimized and calculated, in the same manner as the original compounds, to obtain a set of key descriptor values. Then, these key descriptor values were replaced in the QSAR equation (as *X* variables) to calculate predicted pIC_50_ values of the modified compounds.

## Results and Discussion

### QSAR models

A set of bioactive compounds with DNMT1 inhibitory activity were collected from ChEMBL25 database (EMBL-EBI, 2019[13]) and were preprocessed according to the established protocol (Fourches et al., 2010[18]). A set of curated compounds were divided into 2 groups (i.e., scaffolds A and B) according to their core structures (Figure 1(Fig. 1)). All compounds were optimized and calculated to obtain their descriptor values (as a set of structural representatives). Correlation-based feature selection followed by MLR method were performed to obtain a final set of 6 informative descriptors. Definitions of selected descriptors (Table 1(Tab. 1)) and descriptor values (Supplementary Tables 1-2) of the investigated compounds are provided. Values of selected descriptor together with the bioactivity (pIC_50 _values) were used as input data sets to construct the QSAR models using MLR algorithm. Herein, two QSAR models were separately constructed based on core structure of the compounds (i.e., scaffold A and scaffold B).

For scaffold A, two informative descriptors (i.e., BIC1 and F06[N-O]) were used to construct QSAR model (Equation 2). An influence of each descriptor on pIC_50_ value was demonstrated by its regression coefficient value. The QSAR model revealed that bond information content (BIC1 with regression coefficient = 3.9879) is the most influential descriptor for predictive DNMT1 inhibitory activity of indoles.


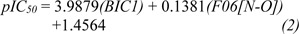


Four selected descriptors were used to build the QSAR model of scaffold B (Equation 3) including electronegativity (R8e and R6e+), van der Waals volume (RDF045v) and topological distance (B09[N-N]) descriptors. R6e+ and B09[N-N]) descriptors had positive effects on the activity of oxazoline and 1,2-oxazole inhibitors as shown by positive regression coefficient values, whereas negative effects were observed for those with negative regression coefficient (i.e., R8e and RDF04v). The R6e+ was shown to be the most influential descriptor with regression coefficient value of 10.1847.


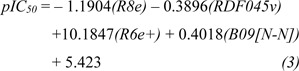


**Figure 3:**
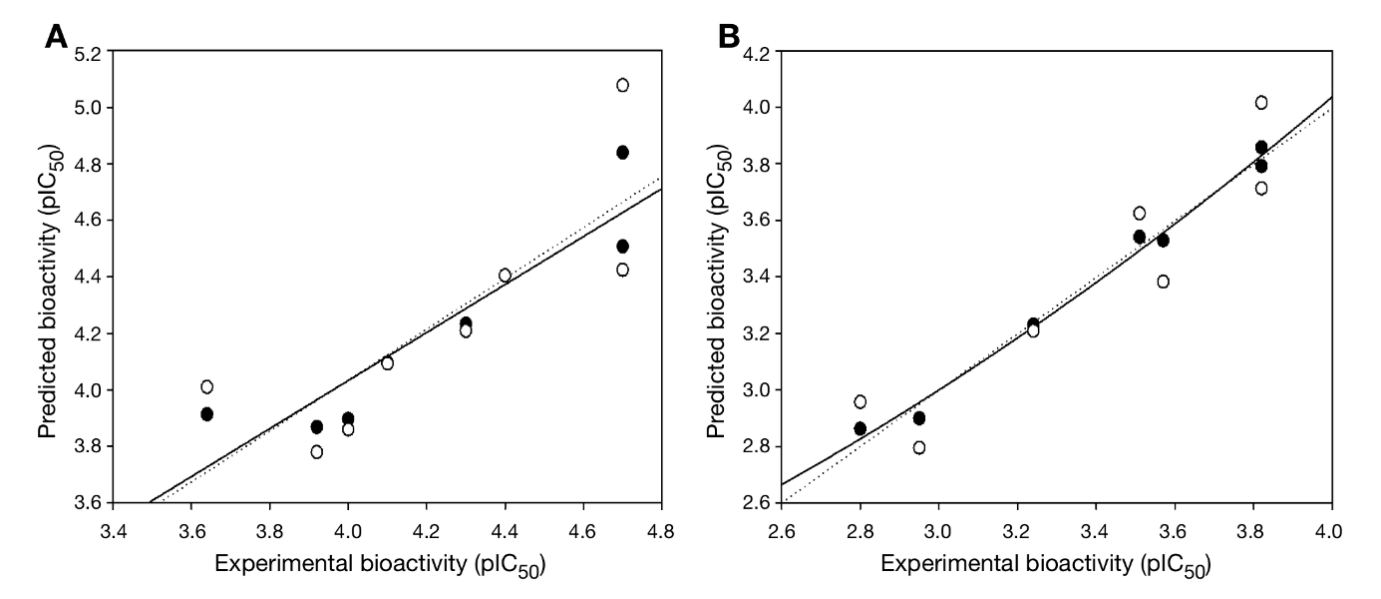
Plots of experimental versus predicted pIC_50_ values of DNMT1 inhibitors generated by QSAR models: (A) scaffold A and (B) scaffold B. Training set: compounds are denoted by black circle and regression line is solid line; LOO-CV Testing set: compounds are denoted by white circle and regression line is dashed line.

In overview, the constructed QSAR models provided acceptable predictive performance, as shown by high R^2^ (0.672-0.988) but low RMSE (0.041-0.224) values. The calculated parameters representing model's performance are summarized in Table 2(Tab. 2). Good predictive performance of the models was observed with low difference between experimental and predicted activities of scaffolds A and B (Table 3(Tab. 3)). Comparative plots of the experimental and predicted pIC_50 _values of the scaffolds A and B are shown in Figure 3(Fig. 3).

### Application of QSAR models for the rational design and prediction of novel DNMT1 inhibitors

**Figure 4:**
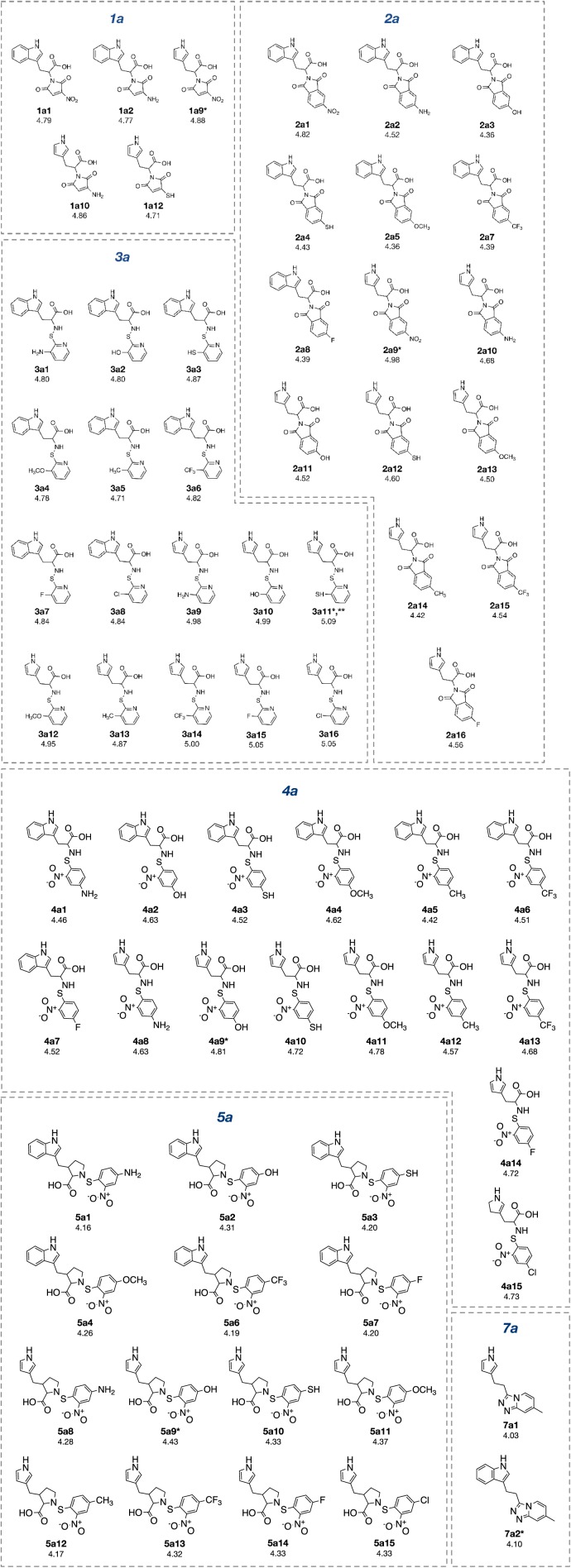
Structurally modified compounds in scaffold A with improved activities (* The most potent compound in the modified subseries, ** The most potent compound of scaffold A).

**Figure 5:**
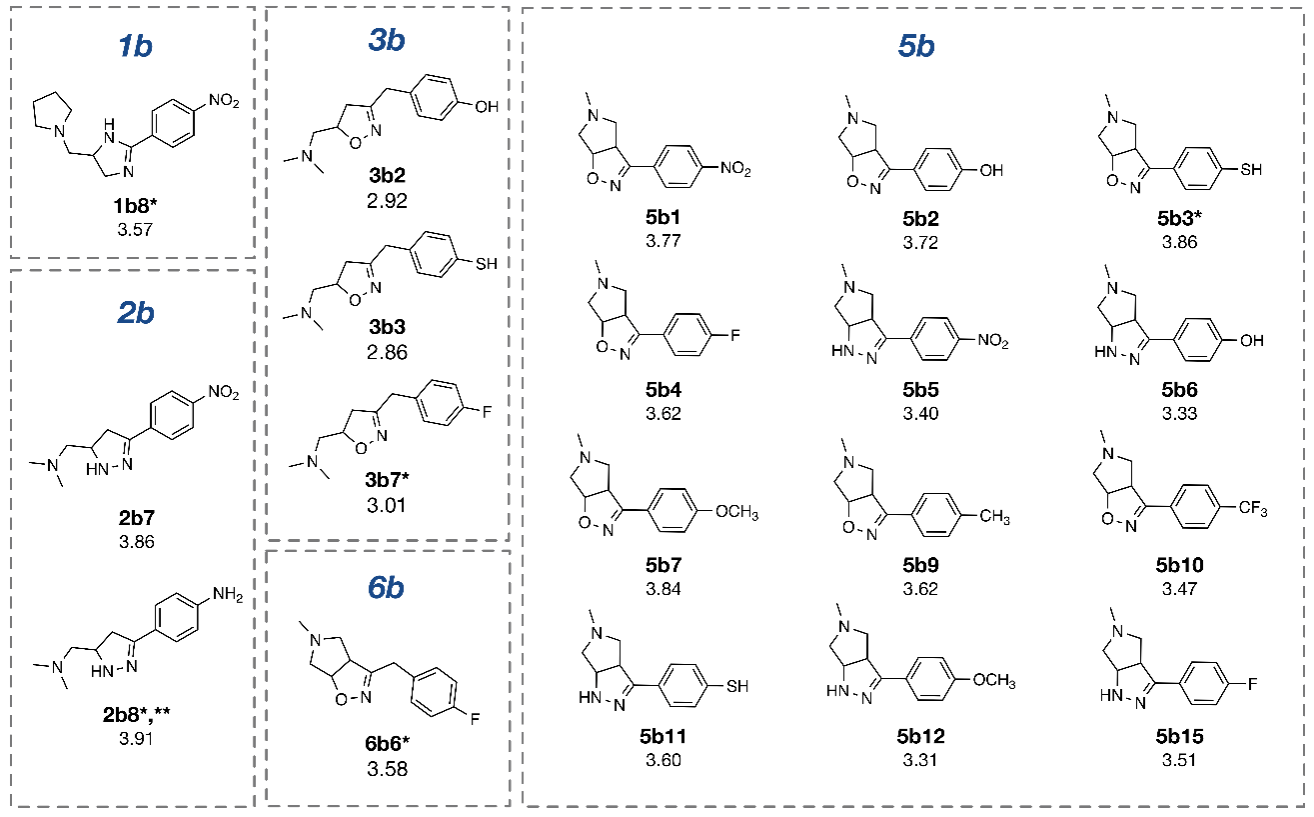
Structurally modified compounds in scaffold B with improved activities (* The most potent compound in the modified subseries, ** The most potent compound of scaffold B).

**Figure 6:**
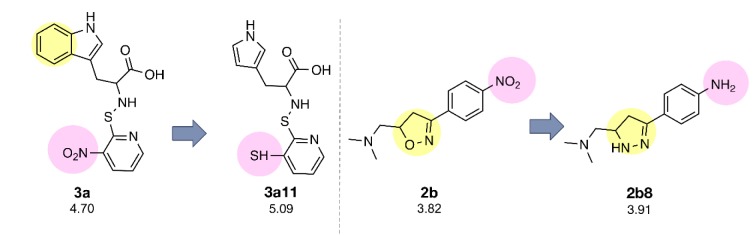
Novel compounds with the most potent predicted activity of scaffolds A (3a11) and B (2b8).

The constructed QSAR models were further applied for the rational design of a novel series of 153 structurally modified compounds with relevant scaffolds. The important descriptors presented in the model were used as a guide for structural modification strategy. Finally, 153 derivatives of scaffolds A (80 modified compounds) and B (73 modified compounds) were virtually designed (Supplementary Figures 1-2), in which their descriptor values were calculated and subsequently applied to the QSAR equations for predicting their activities (Supplementary Tables 3-4). As a result, a series of modified compounds with improved activity (when compared to their parent compounds) are summarized in Figures 4(Fig. 4) and 5(Fig. 5). The promising novel compounds with the most potent predicted activities are highlighted such as compounds **3a11** and **2b8** (Figure 6(Fig. 6)).

### Understanding structure-activity relationship (SAR)

In-depth SAR analysis was performed to consider the important chemical features governing bioactivity of the original scaffolds A and B as well as the modified compounds.

#### Scaffold A

**Figure 7:**
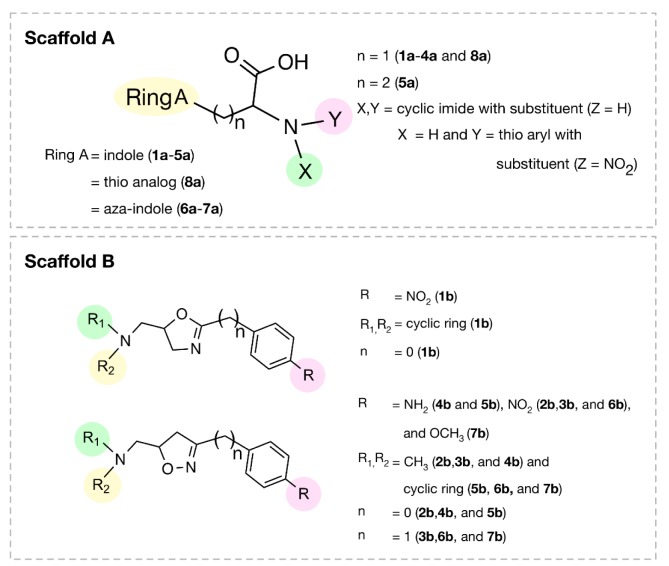
Structural feature of scaffolds A and B

Scaffold A is a series of indole-amino compounds (Figures 1(Fig. 1) and 7(Fig. 7)) which contain ring A (indole and its analogs) substituted by 2-aminocarboxylic acid side chain (**1a**-**5a** and **8a**) and aza-indole ring A (**6a** and **7a**). Bioactivity of these compounds (Table 3(Tab. 3)) was ranked as **1a**~**3a** > **4a** > **2a** > **5a** > **7a** > **6a** > **8a**. Two most potent compounds (**1a** and **3a**) displayed the same activity with pIC_50 _value of 4.70. These two compounds had propanoic acid (three carbon atoms) linker between the indole ring A and 2-amino group (as imide ring for compound **1a**, as aminothiopyridine substituted by 3-NO_2_ group for compound** 3a**). For 2,4-dinitrobenzene analog **4a**, it showed lower activity (pIC_50_= 4.40) when compared with nitropyridine **3a**. Compound **2a** (amino group as fused ring of imide) displayed a lower activity (pIC_50_= 4.30) when compared with the imide ring (**1a**). With the longer linker (four carbon atoms), compound **5a** containing 2-aminobutanoic acid side chain was shown to be less active (pIC_50_= 4.10) when compared to dinitrophenyl compound **4a**. When N atom of the indole ring was replaced by S atom, benzothiophene analog (ring A)** 8a** was obtained with the lowest activity (pIC_50_ = 3.64). Aza-indoles **6a** and **7a** (derived from replacing C atoms in the indole ring by one N and two N atoms, respectively) showed lower activity than the others (**1a**-**5a**), but higher than the thioindole (**8a**).

**Figure 8:**
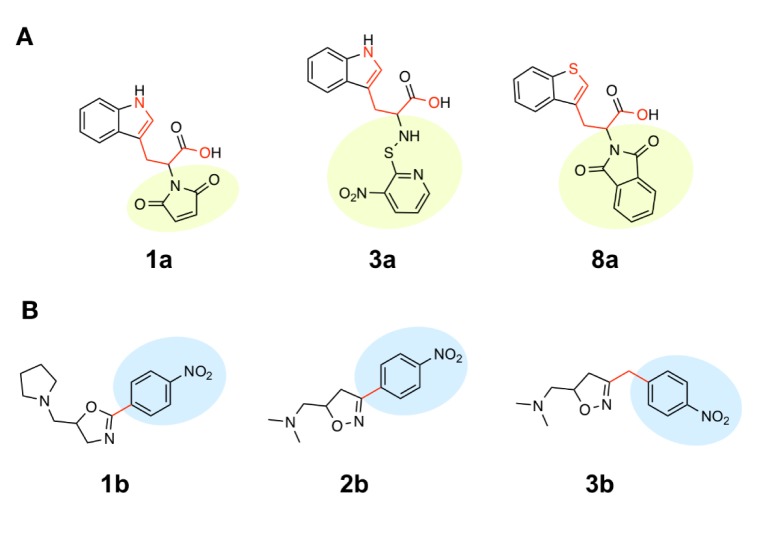
The most potent and least potent compounds in each series. (A) Scaffold A: Two most potent compounds 1a, 3a and the least potent compounds 8a involved in BIC1 (circle color) and F06[N-O] (red color bonds) descriptors; (B) Scaffold B: The most potent compounds 1b, 2b and the least potent compound 3b (red bond indicate carbon linker).

The QSAR model (Equation 1) showed that two descriptors (Supplementary Table 1), BIC1 and F06[N-O] are involved in the SAR. BIC1 is a more influential descriptor than that of F06[N-O] as noted from their regression coefficient values of 3.9879 and 0.1381, respectively. The most potent compounds **1a** and **3a **displayed the highest values of BIC1 (0.696 and 0.710) and of F06[N-O] (2 and 4), respectively compared with the less active compounds i.e., **8a** (B1C1 = 0.616, F06[N-O] = 0). This could be due to the chemical structure (Figure 8A(Fig. 8)) of indole (**3a**) bearing 2-aminopropanoic moiety substituted by thiopyridine, which may display the highest neighborhood symmetry (BIC1 = 0.710) compared with the thioindole (**8a**, BIC1 = 0.616) containing 2-imide propanoic acid. In addition, the indole **3a **constituting propanoic moiety could involve in the highest frequency of N-O at topological distance 6 (F06[N-O] = 4) whereas the thioindole **8a **had the F06[N-O] = 0. Such high topological distance of compound **3a **could be accounted by N atom of indole ring connecting to O atom of carboxylic group as shown in Figure 8A(Fig. 8).

To achieve compounds with more improved activity, core structure or scaffold of the compound and its functional substituents could be modified. For scaffold A (Figures 1(Fig. 1) and 7(Fig. 7)), compounds **1a**-**5a** were structurally modified in which the indole ring A is either conserved or changed to pyrrole ring as well as the 2-amino moiety was substituted by Z group (NO_2_, NH_2_, OH, SH, OCH_3_, CH_3_, CF_3_ and F). The results (Supplementary Figure 1 and Supplementary Table 3) of indole ring and substitution at imide ring by the corresponding Z groups provided derivatives **1a1**-**1a8**. When the indole ring A was changed to pyrrole ring and imide ring was substituted by Z groups, the modified compounds **1a9**-**1a16** were achieved. In **1a** series, most of the compounds showed less potent activity than the parent compound. Pyrrole derivatives with Z= NO_2_ (**1a9**) and NH_2_ (**1a10**) displayed the most improved activity with comparable activity (pIC_50_ = 4.88 and 4.86, respectively) when compared with the indole series (**1a1**, pIC_50_ = 4.79, Z = NO_2_ and **1a2, **pIC_50_ = 4.77, Z = NH_2_). This indicated that NO_2_ group (Z) is the most effective substituent on the imide ring of both indole (**1a1**) and pyrrole (**1a9**) analogs.

Similar results were noted for modified series **2a** (**2a1**-**2a16**), most compounds showed the improved activity, except for compound **2a6**. Both indole (**2a1**) and pyrrole (**2a9**) bearing Z = NO_2_ displayed the most improved activity (pIC_50_ = 4.82 and 4.98, respectively). This indicated that the pyrrole exerted higher activity than the indole. 

In modified compounds **5a** (**5a1** to **5a15**), most compounds showed the improved activity, except for **5a5**. Pyrrole compound (**5a9**) was the most improved one with pIC_50_ = 4.43 whereas the parent compound **5a** showed the pIC_50_ of 4.10.

In case of compounds **3a **and **4a, **all modified compounds (**3a1**-**3a16** and **4a1**-**4a15**) displayed the improved effect. It was shown that aminothiopyridine **3a11** was the most potent modified compound (pIC_50_ = 5.09), which is the pyrrole analog bearing 2-amino moiety substituted by SH (Z) group. In addition, the most improved modified **4a9** (pIC_50_ = 4.81) was pyrrole derivative containing 2-amino moiety substituted by OH (Z) group. Notably, the structurally modification of fused indole ring (**1a**-**5a**) provided the improved compounds as the single ring (pyrrole) compounds. 

Compound **7a** (as bis-triazole condensed ring, pIC_50_ = 4.00) was transformed to pyrrole (**7a1**) and indole (**7a2**) analogs with the remaining one triazole ring. The improved effect was noted for the indole analog **7a2** (pIC_50_ = 4.10). Modified compounds (Supplementary Figure 1) in series **1a**-**5a** and **7a**, mostly showed the improved activity (Supplementary Table 3) when compared with their parent compounds. Particularly, the most potent **3a** (pIC_50_ = 4.70) provided the most improved compound (**3a11**) with the predicted pIC_50_ of 5.09 (BIC1 = 0.772, F06[N-O] = 4). The high predicted pIC_50_ value of 4.98 was observed for compound **2a9** (BIC1 = 0.710, F06[N-O] = 5). In addition, compounds **1a9** and **1a10 **also displayed the improved activity (pIC_50_ =4.88, BIC1 = 0.721, F06[N-O] = 4 and pIC_50_ = 4.86, BIC1 = 0.714, F06[N-O] = 4, respectively). In series **4a**, compound **4a9** was the most improved one (pIC_50_ = 4.81, BIC1 = 0.737, F06[N-O] = 3). Compound **5a9** of series **5a** displayed the most improved effect (pIC_50_ = 4.43, BIC1 = 0.712, F06[N-O] = 1). Compound **7a2** showed slightly improved activity (pIC_50_ = 4.10, BIC1 = 0.663, F06[N-O] = 0) comparing with the parent compound **7a** (pIC_50_ = 4.00, BIC1 = 0.612, F06[N-O] = 0). It should be noted that the most potent modified compound **3a11 **(Figure 6(Fig. 6)) had the highest value of B1C1 = 0.772 and high value of F06[N-O] = 4 when compared with its parent compound (**3a**) as well as with other modified compounds in scaffold A. The highest neighborhood symmetry (BIC1) of compound **3a11 **could be resulted from the single pyrrole (ring A) and a smaller group (Z = SH) on the thiopyridine moiety, which make the molecule even more symmetry than the indole ring A bearing the larger group (Z = NO_2_) of compound **3a**. Structural features of **3a** and **3a11 **(Figure 6(Fig. 6)) showed that these compounds had the same value of F06[N-O] = 4 representing by 6 bonds, which are a part of linkage between N atom (indole/pyrrole rings) and O atom of carboxylic moiety, whereas such property was not seen in the thioindole **8a **(Figure 8A(Fig. 8)).

#### Scaffold B

Scaffold B is a series of bioactive 1,2-oxazoles (Figures 1(Fig. 1) and 7(Fig. 7)) with 3,5-disubstitution pattern including compounds **2b**-**7b, **whereas compound **1b** represents oxazoline. It was found that both nitro analog (R = NO_2_) of oxazoline **1b **and oxazole **2b **exerted the highest bioactivity (pIC_50_ = 3.82). On the other hand, nitro compound **3b** with one carbon linker (n=1) between 1,2-oxazole and phenyl group displayed the lowest activity (pIC_50_ = 2.80). These compounds (**1b**, **2b** and **3b**) are shown in Figure 8B(Fig. 8). Amino (R = NH_2_) compound **4b** (pIC_50_ = 3.57) showed weaker activity when compared with its nitro analog (**2b**). Nitro compound **6b** (R = NO_2_, pIC_50_ = 3.51) exhibited higher activity than amino compound **5b** (pIC_50_ = 3.24). With the same core scaffold, methoxy compound (R = OCH_3_, **7b**) displayed lower activity (pIC_50_ = 2.95) when compared with the nitro compound **6b**. Bioactivities of these compounds (Table 3(Tab. 3)) are ranked as followed:** 1b **~** 2b **>** 4b **>** 6b **>** 5b **>** 7b **>** 3b**.

The QSAR study (Equation 2) showed that the two most potent compounds **1b** and **2b** displayed significant descriptors (Supplementary Table 2) with low values of R8e = 0.356, RDF045v = 4.874, but high values of R6e+ = 0.035, B09[N-N] = 1 and with low values of R8e = 0.437, RDF045v = 4.693, but high values of R6e+ = 0.031, B09[N-N] = 1, respectively. On the other hand, the least active compound **3b** displayed the high values of R8e = 0.516 and RDF045v = 5.677, but the low values of R6e+ = 0.026, B09[N-N] = 0. It should be noted that the oxazole **2b **had the lowest van der Waals volume (RDF045v = 4.693) and low value of electronegativity (R8e = 0.437) when compared with the oxazole **3b **having the highest van der Waals volume (RDF045v = 5.677) and high electronegativity (R8e = 0.516). The highest RDF045v value of compound **3b **could be due to the presence of CH_2_ group (n = 1, Figure 8B(Fig. 8)) linking between the oxazole and nitro phenyl rings whereas the compound without CH_2_ group had the lowest van der Waals volume as noted for the most potent compound **2b**. The improved activity of scaffold B compounds was performed (Supplementary Figure 2 and Supplementary Table 4) as followed.

Oxazoline **1b** was structurally modified by replacing NO_2_ (R) group with various substituents (i.e., NH_2_, OH, SH, OCH_3_, CH_3_, CF_3_ and F) to obtain compounds **1b1**-**1b7**. When O atom in the oxazoline scaffold was replaced by N atom, a new imidazole core was achieved as shown by derivatives **1b8**-**1b15**. The imidazole **1b8** (R = NO_2_) was shown to be the most potent one. However, all of these modified compounds **1b1**-**1b15** displayed lower activity (pIC_50_ = 2.79-3.57) than their parent compound (**1b**, pIC_50_ = 3.82).

Oxazole **2b **was similarly modified to give compounds **2b1**-**2b14 **(pIC_50_ = 2.92-3.91), in which 1,2 diazole **2b8 **(R = NH_2_) was shown to be the most improved compound (pIC_50_ = 3.91) when compared with the parent compound **2b** (pIC_50_ = 3.82)

R group (NO_2_) of **3b** and its oxazole scaffold were transformed as mentioned above to obtain compound **3b1**-**3b15**. The results showed that oxazole compound **3b7 **(R = F) exerted improved activity (pIC_50_ = 3.01) comparing to the parent compound **3b** (pIC_50_ = 2.80).

For compound **5b**, its oxazole scaffold and R group were structurally modified to afford compounds **5b1**-**5b15** (pIC_50_ = 2.92-3.86). Compound **5b3 **(R = SH) exerted the most improved activity (pIC_50_ = 3.86) when compared with its parent (**5b**, R = NH_2_, pIC_50_ = 3.24). 

Similarly, compound **6b **(pIC_50_ = 3.51) was modified as described for compound **5b **to obtain compounds **6b1**-**6b14 **(pIC_50_ = 2.40-3.58). It was found that compound **6b6** (R = F) displayed the most improved DNMT1 inhibitory effect with the predicted pIC_50_ value of 3.58 when compared with the parent **6b** (R = NO_2_).

Notably, the most improved compound **2b8 **displayed the lower values of R8e = 0.421 and RDF045v = 4.547, but higher value of R6e+ = 0.035 as compared to the parent compound **2b **(R8e = 0.437, RDF045v = 4.693 and R6e+ = 0.031). The lower van der Waals volume (RDF045v) and lower electronegativity (R8e) of the most improved compound **2b8 **(Figure 6(Fig. 6)) could be due to the smaller size and less electronegativity of the NH_2_ (R) group comparing with the nitro group of its parent **2b**.

### Chemical space of the studied compounds

Not only potent bioactivity but also drug-like properties of the compounds are essential for successful drug development. Chemical space exploration is a method to investigate the drug-likeness of the compounds in which the Lipinski 'rule of five' is used as a guideline to determine drug-like properties (Reymond and Awale, 2012[47]). The chemical space plots of the inhibitors and the related discussion are provided in Supplementary Information (Supplementary Figures 3-4). It was demonstrated that most of the investigated compounds were distributed within a space of the Lipinski 'rule of five', which indicate their potential to be further developed as drugs.

## Conclusion

Current attention has been given to the epigenetic targets due to their modifiable nature throughout lifetime. Among these targets, DNMT1 is a promising target which plays roles in many diseases. In this study, QSAR modeling of indole-based and oxazoline/oxazole-based DNMT1 inhibitors was performed along with in-depth SAR analysis. Two models were successfully constructed providing good predictive performance. A set of key structural features influencing the DNMT1 inhibitory effect of the compounds were revealed such as bond information, frequency of [N-O], electronegativity, van der Waals volume and topological distance. To increase structural diversity, the QSAR findings were further applied as a guide for *in silico* structural modification to design a set of 153 novel inhibitors and their activities were predicted. Finally, a set of promising newly designed inhibitors were highlighted to be further developed as potential DNMT1 inhibitors for therapeutics. In summary, this study demonstrates the facilitating role of QSAR modeling toward effective drug development in terms of rational design, screening and structural optimization. However, further synthesis of promising modified inhibitors and experimental studies are required to confirm DNMT1 inhibitory activity.

## Supplementary information

Supplementary information is available on the EXCLI Journal website.

## Conflict of interest

The authors declare that they have no conflict of interest.

## Acknowledgements

This work is supported by the Royal Golden Jubilee Ph.D. Scholarship (No. PHD00502558), the annual budget grant (B.E. 2562-2563) of Mahidol University and TRF Research Career Development Grant (No. RSA6280075) from the Thailand Research Fund of the Office of Higher Education Commission and Mahidol University. The authors would like to thank Dr. Nuttapat Anuwongcharoen for optimization of the compounds.

## Supplementary Material

Supplementary information

## Figures and Tables

**Table 1:**
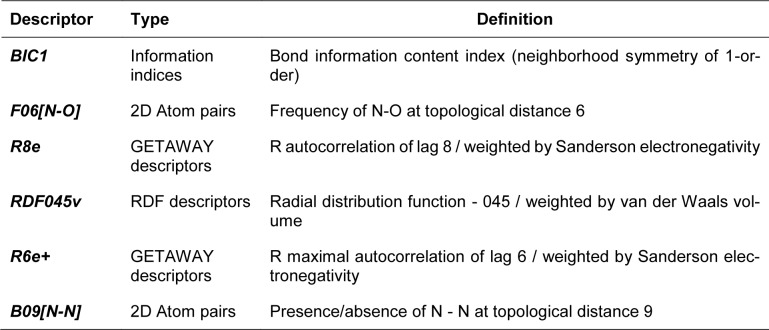
Definition of informative descriptors for QSAR modeling

**Table 2:**
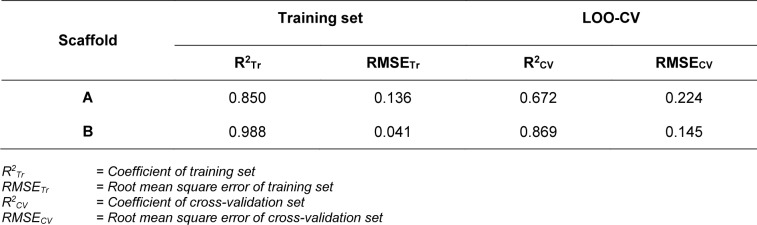
Summary of predictive performance of QSAR models

**Table 3:**
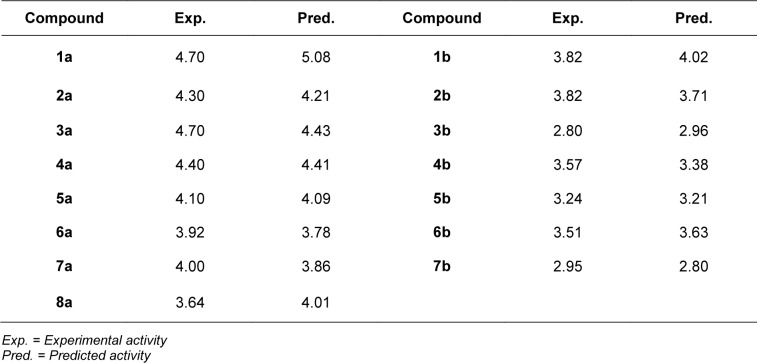
Experimental and predicted bioactivities (pIC_50_) of scaffolds A and B
