# Supplementary material for: QSAR-driven rational design of novel DNA methyltransferase 1 inhibitors
Source: EXCLI J. 2020 Apr 2;19:458–75. doi: 10.17179/excli2020-1096 (PMC7214779; doi:10.17179/excli2020-1096)
Supplement: Supplementary information [file EXCLI-19-458-s-001.pdf]

EXCLI Journal 2020;19:458-475 – ISSN 1611-2156
Received: January 31, 2020, accepted: March 24, 2020, published: April 02, 2020

Supplementary information to:

QSAR-DRIVEN RATIONAL DESIGN OF NOVEL DNA METHYLTRANSFERASE 1 INHIBITORS

Chuleeporn Phanus-umporn[1], Veda Prachayasittikul[1,*], Chanin Nantasenamat[1], Supaluk Prachayasittikul[1], Virapong Prachayasittikul[2]

[1] Center of Data Mining and Biomedical Informatics, Faculty of Medical Technology, Mahidol University, Bangkok 10700, Thailand
[2] Department of Clinical Microbiology and Applied Technology, Faculty of Medical Technology, Mahidol University, Bangkok 10700, Thailand

* **Corresponding author:** Veda Prachayasittikul, Center of Data Mining and Biomedical Informatics, Faculty of Medical Technology, Mahidol University, Bangkok 10700, Thailand. Phone: +66 2 441 4371; Fax: +66 2 441 4380; E-mail: veda.pra@mahidol.ac.th

http://dx.doi.org/10.17179/excli2020-1096

This is an Open Access article distributed under the terms of the Creative Commons Attribution License (http://creativecommons.org/licenses/by/4.0/).

Supplementary Table 1: Descriptors and experimental pIC_{50} values of scaffold A

COMPOUND	BIC1	F06[N-O]	pIC_{50}
**1a**	0.696	2	4.70
**2a**	0.627	2	4.30
**3a**	0.710	4	4.70
**4a**	0.670	2	4.40
**5a**	0.662	0	4.10
**6a**	0.570	1	3.92
**7a**	0.612	0	4.00
**8a**	0.616	0	3.64

Received: January 31, 2020, accepted: March 24, 2020, published: April 02, 2020

Supplementary Table 2: Descriptors and experimental pIC_{50} values of scaffold B

COMPOUND	R8E	RDF045V	R6E+	B09[N-N]	pIC_{50}
**1b**	0.356	4.874	0.035	1	3.82
**2b**	0.437	4.693	0.031	1	3.82
**3b**	0.516	5.677	0.026	0	2.80
**4b**	0.396	5.467	0.030	1	3.57
**5b**	0.467	4.986	0.03	0	3.24
**6b**	0.487	5.184	0.031	1	3.51
**7b**	0.528	5.624	0.029	0	2.95

Supplementary Table 3: Descriptors and predicted pIC_{50} values of modified DNMT1 inhibitors of scaffold A

COMPOUNDS	BIC1	F06[N-O]	PREDICTED pIC_{50}
**1a1**	0.697	4	4.79
**1a2**	0.692	4	4.77
**1a3**	0.705	2	4.54
**1a4**	0.715	2	4.58
**1a5**	0.682	2	4.45
**1a6**	0.672	2	4.41
**1a7**	0.711	2	4.57
**1a8**	0.706	2	4.55
**1a9***	0.721	4	4.88
**1a10**	0.714	4	4.86
**1a11**	0.733	2	4.66
**1a12**	0.746	2	4.71
**1a13**	0.699	2	4.52
**1a14**	0.688	2	4.48
**1a15**	0.737	2	4.67
**1a16**	0.736	2	4.67
**2a1**	0.671	5	4.82
**2a2**	0.665	3	4.52
**2a3**	0.659	2	4.36
**2a4**	0.676	2	4.43
**2a5**	0.660	2	4.36
**2a6**	0.642	2	4.29
**2a7**	0.666	2	4.39
**2a8**	0.667	2	4.39
**2a9***	0.710	5	4.98
**2a10**	0.704	3	4.68
**2a11**	0.698	2	4.52
**2a12**	0.720	2	4.60
**2a13**	0.694	2	4.50
**2a14**	0.674	2	4.42
**2a15**	0.704	2	4.54
**2a16**	0.710	2	4.56

EXCLI Journal 2020;19:458-475 – ISSN 1611-2156
Received: January 31, 2020, accepted: March 24, 2020, published: April 02, 2020

Supplementary Table 3 (cont.): Descriptors and predicted pIC_{50} values of modified DNMT1 inhibitors of scaffold A

COMPOUNDS	BIC1	F06[N-O]	PREDICTED pIC_{50}
**3a1**	0.699	4	4.80
**3a2**	0.699	4	4.80
**3a3**	0.718	4	4.87
**3a4**	0.696	4	4.78
**3a5**	0.678	4	4.71
**3a6**	0.705	4	4.82
**3a7**	0.709	4	4.84
**3a8**	0.709	4	4.84
**3a9**	0.746	4	4.98
**3a10**	0.748	4	4.99
**3a11*,***	0.772	4	5.09
**3a12**	0.738	4	4.95
**3a13**	0.718	4	4.87
**3a14**	0.751	4	5.00
**3a15**	0.763	4	5.05
**3a16**	0.763	4	5.05
**4a1**	0.684	2	4.46
**4a2**	0.692	3	4.63
**4a3**	0.700	2	4.52
**4a4**	0.690	3	4.62
**4a5**	0.673	2	4.42
**4a6**	0.697	2	4.51
**4a7**	0.700	2	4.52
**4a8**	0.726	2	4.63
**4a9***	0.737	3	4.81
**4a10**	0.748	2	4.72
**4a11**	0.729	3	4.78
**4a12**	0.711	2	4.57
**4a13**	0.740	2	4.68
**4a14**	0.750	2	4.72
**4a15**	0.752	2	4.73
**5a1**	0.678	0	4.16
**5a2**	0.680	1	4.31
**5a3**	0.687	0	4.20
**5a4**	0.669	1	4.26
**5a5**	0.655	0	4.07
**5a6**	0.686	0	4.19
**5a7**	0.687	0	4.20
**5a8**	0.709	0	4.28
**5a9***	0.712	1	4.43
**5a10**	0.721	0	4.33
**5a11**	0.696	1	4.37
**5a12**	0.680	0	4.17
**5a13**	0.717	0	4.32
**5a14**	0.721	0	4.33
**5a15**	0.721	0	4.33
**7a1**	0.645	0	4.03
**7a2***	0.663	0	4.10

*The most potent compounds in subseries, ** The most potent compound of scaffold A

EXCLI Journal 2020;19:458-475 – ISSN 1611-2156
Received: January 31, 2020, accepted: March 24, 2020, published: April 02, 2020

Supplementary Table 4: Descriptors and predicted pIC_{50} values of modified DNMT1 inhibitors of scaffold B

COMPOUND	R8E	RDF045V	R6E+	B09[N-N]	PREDICTED pIC_{50}
**1b1**	0.338	6.513	0.036	1	3.25
**1b2**	0.354	5.660	0.035	0	3.15
**1b3**	0.334	5.494	0.036	0	3.25
**1b4**	0.349	6.244	0.033	0	2.91
**1b5**	0.328	6.576	0.034	0	2.82
**1b6**	0.359	6.261	0.032	0	2.88
**1b7**	0.368	5.875	0.037	0	3.07
**1b8***	0.477	4.922	0.023	1	3.57
**1b9**	0.450	5.760	0.023	1	3.28
**1b10**	0.448	5.274	0.022	0	3.06
**1b11**	0.432	5.102	0.022	0	3.15
**1b12**	0.449	5.540	0.021	0	2.94
**1b13**	0.456	5.908	0.022	0	2.80
**1b14**	0.512	5.761	0.022	0	2.79
**1b15**	0.444	5.064	0.024	0	3.17
**2b1**	0.372	5.055	0.031	0	3.33
**2b2**	0.364	4.818	0.032	0	3.44
**2b3**	0.367	5.284	0.029	0	3.22
**2b4**	0.424	5.665	0.029	0	3.01
**2b5**	0.510	5.600	0.028	0	2.92
**2b6**	0.345	4.887	0.031	0	3.42
**2b7**	0.481	4.431	0.033	1	3.86
**2b8*,****	0.421	4.547	0.035	1	3.91
**2b9**	0.397	4.889	0.034	0	3.39
**2b10**	0.388	4.675	0.035	0	3.50
**2b11**	0.406	5.202	0.034	0	3.26
**2b12**	0.459	5.49	0.034	0	3.08
**2b13**	0.553	5.321	0.033	0	3.03
**2b14**	0.363	4.616	0.035	0	3.55
**3b1**	0.494	6.286	0.026	0	2.65
**3b2**	0.500	5.639	0.028	0	2.92
**3b3**	0.482	5.813	0.027	0	2.86
**3b4**	0.574	6.204	0.026	0	2.59
**3b5**	0.490	6.606	0.027	0	2.54
**3b6**	0.517	5.937	0.027	0	2.77
**3b7***	0.508	5.567	0.035	0	3.01
**3b8**	0.559	5.935	0.025	0	2.70
**3b9**	0.543	7.075	0.031	0	2.34
**3b10**	0.540	6.619	0.026	0	2.47
**3b11**	0.524	6.085	0.032	0	2.75
**3b12**	0.582	6.839	0.030	0	2.37
**3b13**	0.542	7.217	0.031	0	2.28
**3b14**	0.592	7.406	0.033	0	2.17
**5b1**	0.504	3.457	0.029	0	3.77
**5b2**	0.446	3.781	0.030	0	3.72
**5b3***	0.424	3.538	0.031	0	3.86
**5b4**	0.418	4.081	0.028	0	3.62
**5b5**	0.481	4.419	0.027	0	3.40
**5b6**	0.579	4.309	0.027	0	3.33
**5b7**	0.430	3.598	0.032	0	3.84
**5b8**	0.465	5.274	0.027	0	3.09
**5b9**	0.493	3.887	0.029	0	3.62
**5b10**	0.460	4.388	0.030	0	3.47
**5b11**	0.428	4.164	0.030	0	3.60
**5b12**	0.442	4.803	0.028	0	3.31
**5b13**	0.493	5.560	0.025	0	2.92

EXCLI Journal 2020;19:458-475 – ISSN 1611-2156
Received: January 31, 2020, accepted: March 24, 2020, published: April 02, 2020

Supplementary Table 4 (cont.): Descriptors and predicted pIC_{50} values of modified DNMT1 inhibitors of scaffold B

COMPOUND	R8E	RDF045V	R6E+	B09[N-N]	PREDICTED pIC_{50}
**5b14**	0.549	4.786	0.026	0	3.17
**5b15**	0.435	4.399	0.031	0	3.51
**6b1**	0.478	5.717	0.030	1	3.33
**6b2**	0.494	4.487	0.032	0	3.41
**6b3**	0.477	4.450	0.031	0	3.44
**6b4**	0.466	5.789	0.030	0	2.92
**6b5**	0.493	4.859	0.030	0	3.25
**6b6***	0.518	4.131	0.038	0	3.58
**6b7**	0.520	5.652	0.030	1	3.31
**6b8**	0.504	6.993	0.029	1	2.80
**6b9**	0.525	6.067	0.031	0	2.75
**6b10**	0.504	6.29	0.03	0	2.68
**6b11**	0.529	6.788	0.026	0	2.41
**6b12**	0.496	7.004	0.029	0	2.40
**6b13**	0.53	6.523	0.023	0	2.48
**6b14**	0.549	5.802	0.033	0	2.85

*The most potent compounds in subseries, ** The most potent compound of scaffold B

EXCLI Journal 2020;19:458-475 – ISSN 1611-2156
Received: January 31, 2020, accepted: March 24, 2020, published: April 02, 2020

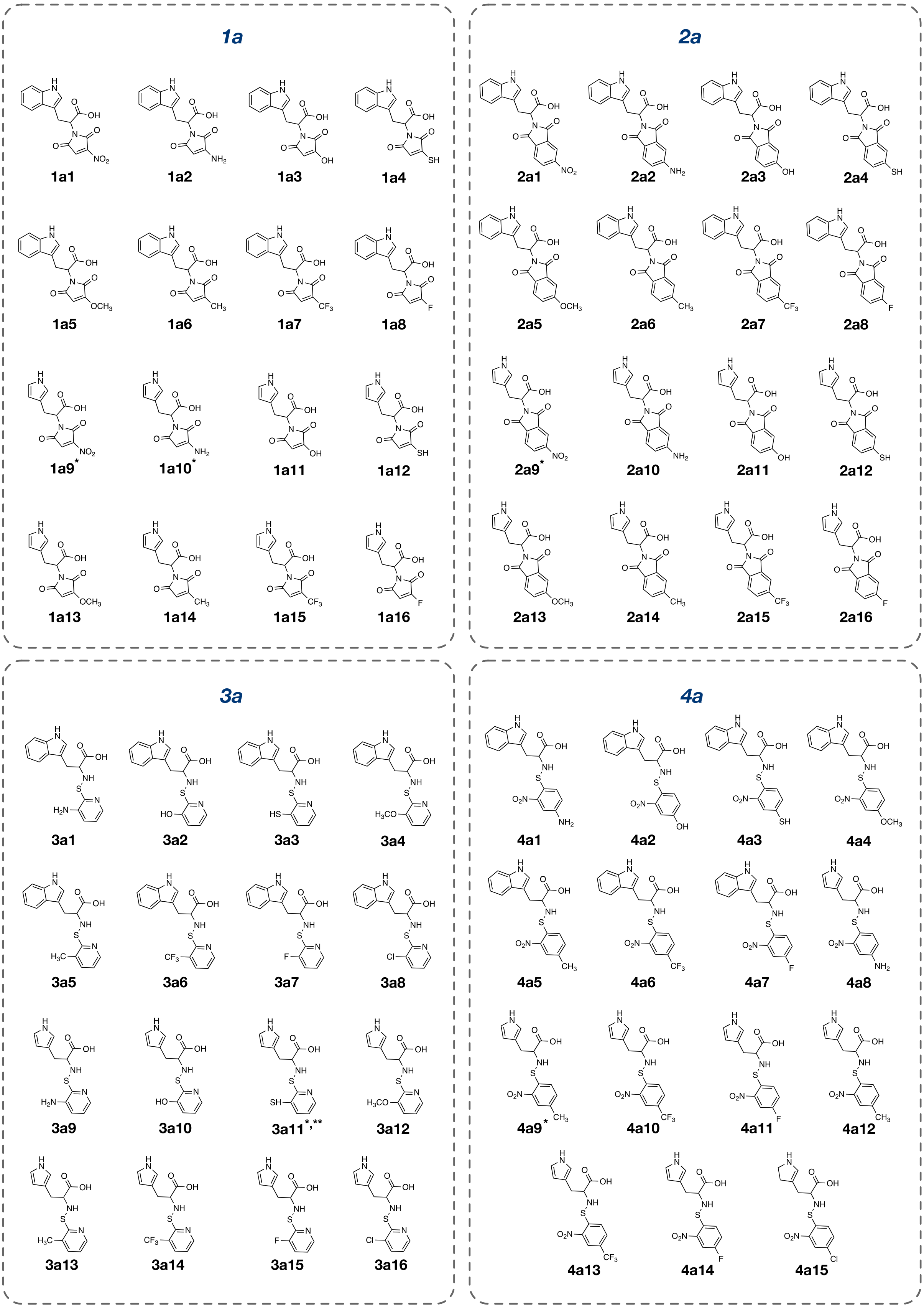

Supplementary Figure 1: Structurally modified compounds **1a-4a** (* The most potent compounds in the modified subseries, ** The most potent compound of scaffold A)

EXCLI Journal 2020;19:458-475 – ISSN 1611-2156
Received: January 31, 2020, accepted: March 24, 2020, published: April 02, 2020

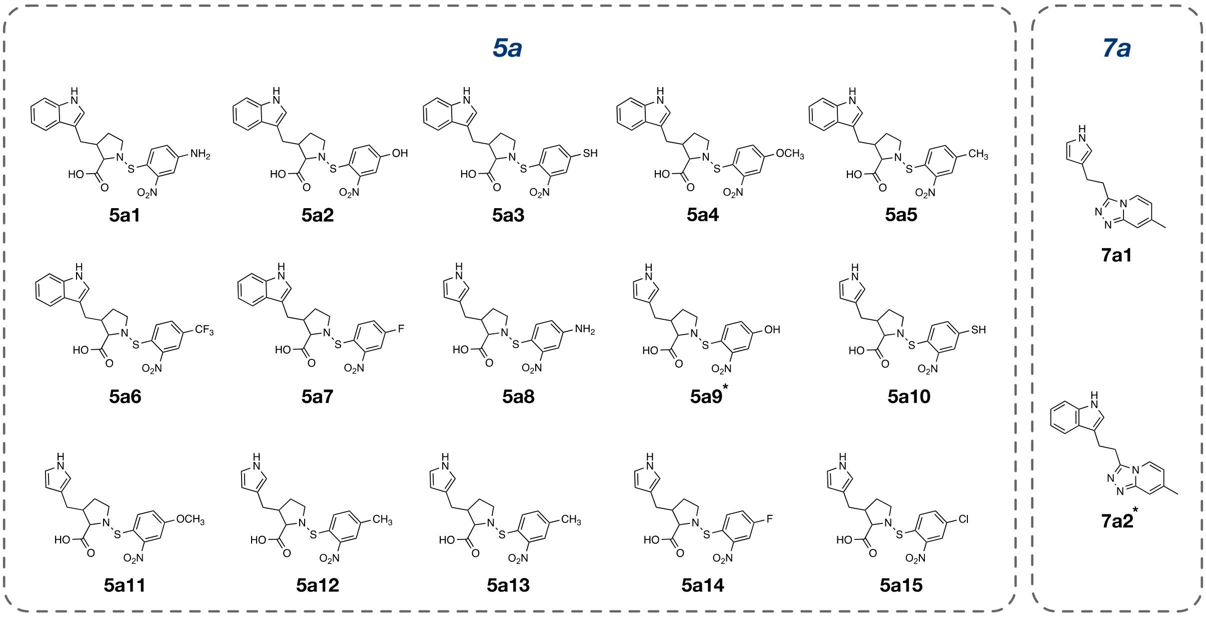

Supplementary Figure 1 (cont.): Structurally modified compounds **5a** and **7a** (* The most potent compounds in the modified subseries, ** The most potent compound of scaffold A)

EXCLI Journal 2020;19:458-475 – ISSN 1611-2156
Received: January 31, 2020, accepted: March 24, 2020, published: April 02, 2020

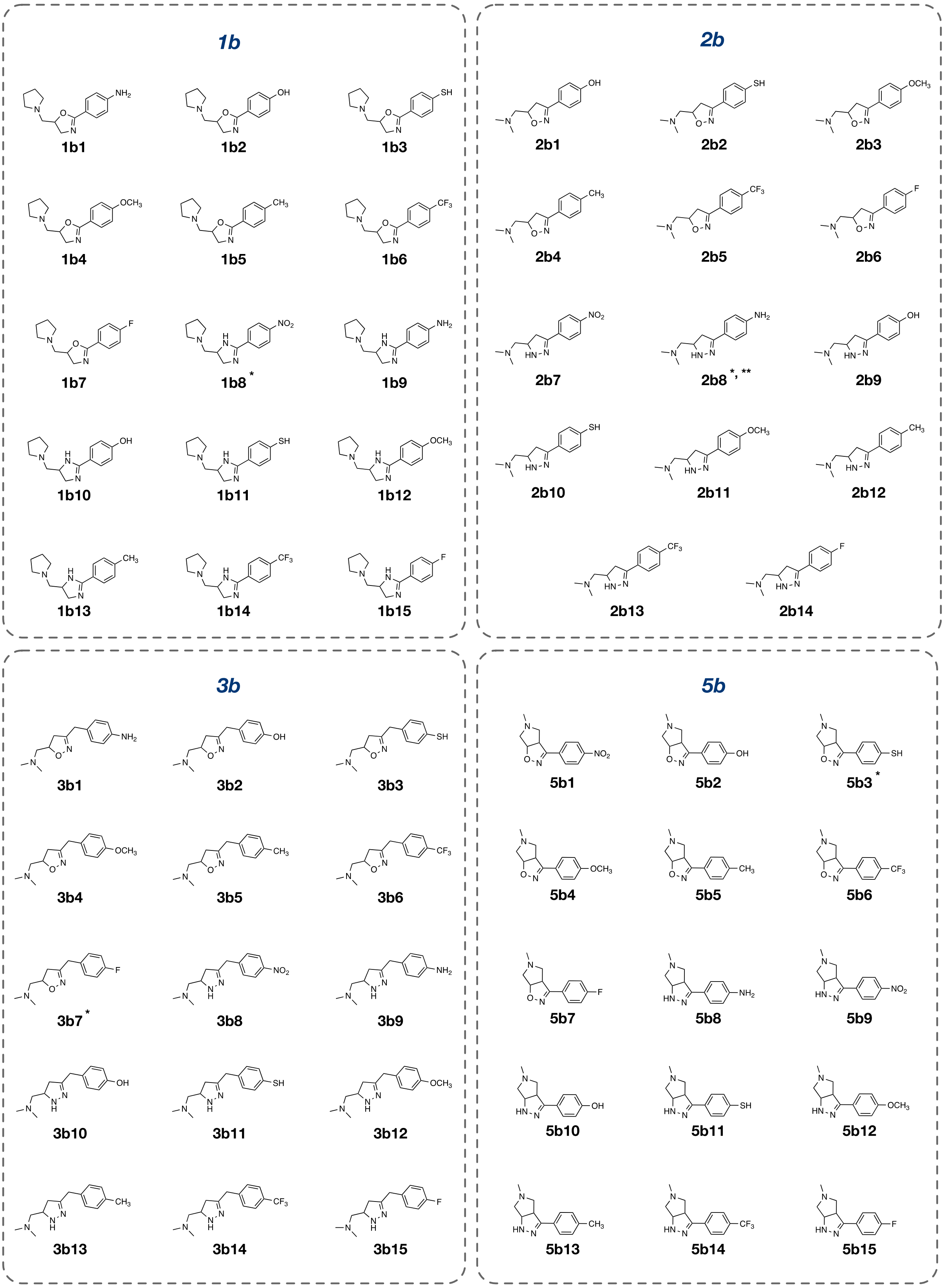

Supplementary Figure 2: Structurally modified compounds **1b-3b** and **5b** (* The most potent compounds in the modified subseries, ** The most potent compound of scaffold B)

EXCLI Journal 2020;19:458-475 – ISSN 1611-2156
Received: January 31, 2020, accepted: March 24, 2020, published: April 02, 2020

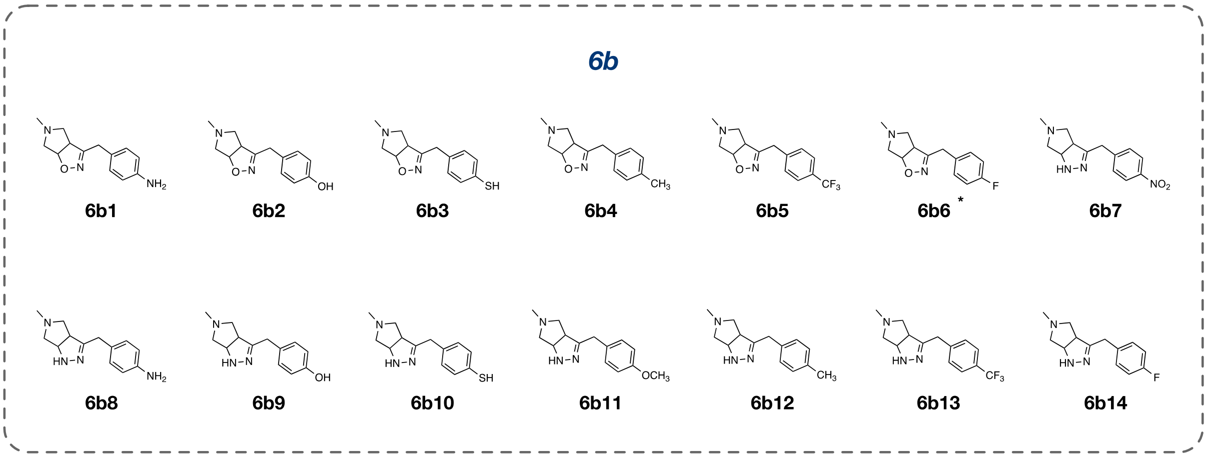

Supplementary Figure 2 (cont.): Structurally modified compounds **6b** (* The most potent compounds in the modified subseries, ** The most potent compound of scaffold B)

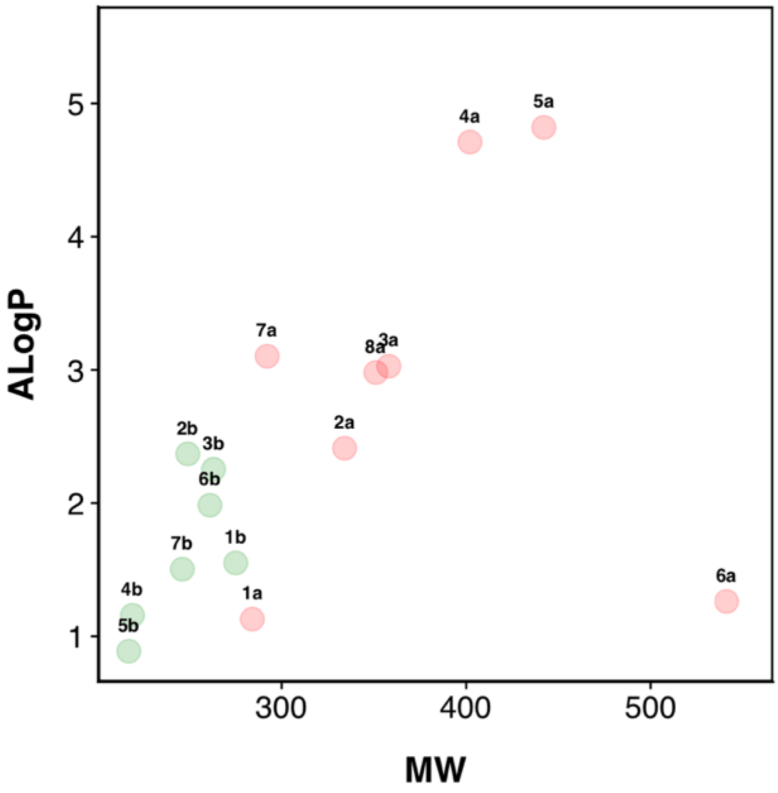

Supplementary Figure 3: Plot of MW versus ALogP of scaffolds A and B (15 compounds), red color represents scaffold A. Green color represents scaffold B.

EXCLI Journal 2020;19:458-475 – ISSN 1611-2156
Received: January 31, 2020, accepted: March 24, 2020, published: April 02, 2020

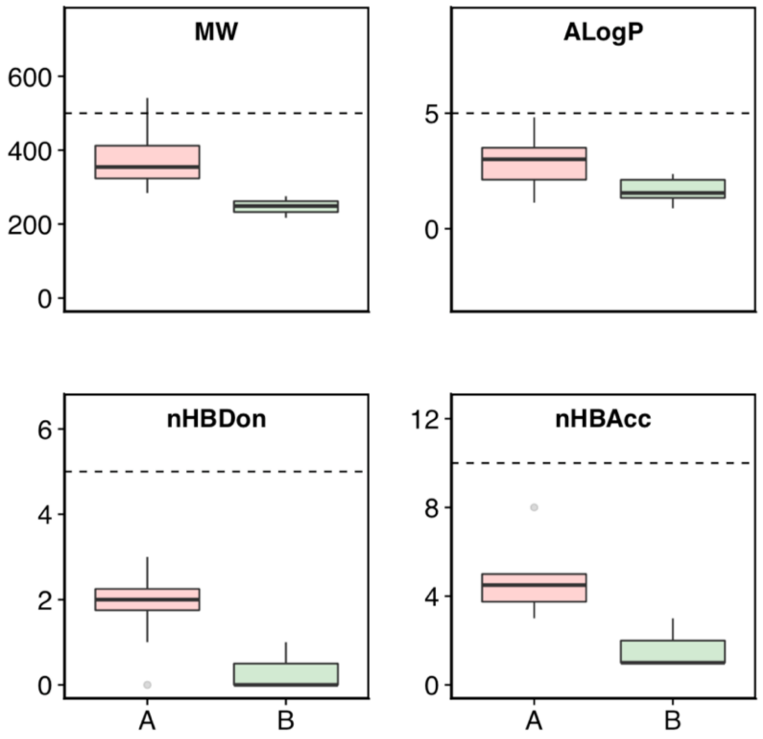

Supplementary Figure 4: Distribution of Lipinski's rule of five, scaffold A (red) and scaffold B (green)

Chemical space of DNMT1 inhibitors

Chemical space has become a key concept in drug discovery and development. It was performed to explore the property of chemical structures by analyzing a set of Lipinski 'rule of five'. The Lipinski 'rule of five' is a guideline of drug-likeness that would make it a likely orally active drug in humans comprising the molecular weight (MW; < 50), Ghose–Crippen–Viswanadhan octanol-water partition coefficient (ALogP; < 5), number of hydrogen bond donors (nHBDon; > 5) and number of hydrogen bond acceptors (nHBAcc; > 10) (Lipinski et al., 2001). MW represents the molecular mass of compounds that is commonly used because it can easily be interpreted and calculated the appropriate size of compounds for its passage via lipid membrane. ALogP is the most widely used method for estimating the lipophilicity of compounds, which implements the potency of distribution and elimination of a drug in the body. nHBDon and nHBAcc represent hydrogen bonding ability. Visualization of the chemical space i.e. MW of scaffolds A and B as the function of AlogP is shown in Supplementary Figure 3. A distribution of most compounds was observed within the space of MW less than 500 and AlogP less than 5. There was only one compound (**6a**) amongst the scaffold A, which was noted out of the Lipinski's rule showing higher MW > 500. This could be due to its complex chemical structure bearing many ring systems and substituents (Figure 1). While, scaffold B was densed distribution of MW less than 300 and AlogP less than 3. Thus, it was revealed that the scaffold A represents a diverse MW of ALogP whereas scaffold B represents the MW and ALogP in a range of good absorption, moderate solubility, but low permeability and metabolism (Stocks, 2013). In addition, a visualization of the distribution results of the Lipinski's descriptors are shown as box plots in Supplementary Figure 4. Analysis of the box plots revealed that the average of all Lipinski's value of scaffold A was shown to be higher than scaffold B. Additionally,

the capacity of hydrogen bonding of scaffold A was higher than scaffold B. It can be implied that the scaffold A provided higher hydrogen bonding ability than the scaffold B.

Based on the QSAR results, the most improved DNMT1 inhibitors (Figure 5) require the molecules with high neighborhood symmetry (BIC1) and high frequency of N-O at topological distance 6 (F06[N-O]) as demonstrated by the pyrrole compound **3a11** of scaffold A and by the pyrazole **2b8** containing aminophenyl substituent with smaller size/van der Waals volume (RDF045v) and less electronegativity (R8e) of scaffold B. In addition, chemical structures of scaffold A bearing more substituents/ring systems with electron donor and electron acceptor atoms (N, O and S), which are capable of forming H-bonding compared with the structures of scaffold B (Figure 1). This shows a good correlation of scaffold A with the H-bonding ability described by Lipinski's rule.

REFERENCES

Lipinski CA, Lombardo F, Dominy BW, Feeney PJ. Experimental and computational approaches to estimate solubility and permeability in drug discovery and development settings. Adv Drug Deliv Rev. 2001;46(1–3):3–26.

Stocks M. The small molecule drug discovery process – from target selection to candidate selection. In: Ganellin R, Roberts S, Jefferis R (eds.): Introduction to biological and small molecule drug research and development (pp 81–126). Amsterdam: Elsevier, 2013.
